# Toward handheld optically guided biopsy combining diffuse reflectance spectroscopy and autofluorescence

**DOI:** 10.1117/1.JBO.30.5.057001

**Published:** 2025-05-06

**Authors:** Lotte M. de Roode, Simon T. Sørensen, Stefan D. van der Stel, Lisanne L. de Boer, Yineng Wang, Huihui Lu, Stefan Andersson-Engels, Theo J. M. Ruers, Ray Burke

**Affiliations:** a Netherlands Cancer Institute-Antoni van Leeuwenhoek, Amsterdam, The Netherlands; b Tyndall National Institute, University College Cork, IPIC, Biophotonics@Tyndall, Cork, Ireland; c University College Cork, Department of Physics, Cork, Ireland

**Keywords:** autofluorescence, diffuse reflectance spectroscopy, cancer detection, miniaturizing, optical biopsy

## Abstract

**Significance:**

We aim to validate the technologies essential for a handheld, optically guided biopsy device designed to enhance the diagnostic yield and accuracy of percutaneous liver biopsy procedures.

**Aim:**

We aim to combine diffuse reflectance spectroscopy (DRS) and autofluorescence (AF) spectroscopy in a fiber-optic needle probe using mini spectrometers for the classification of tumor and healthy tissues.

**Approach:**

A fiber-optic needle probe combining DRS and AF spectroscopy, incorporating mini spectrometers to facilitate future integration into a biopsy actuator, was designed and built. This custom probe was used to measure healthy liver tissues and colorectal metastases in excised liver segments. A linear discriminant analysis was applied to the DRS and AF data to distinguish tumors from healthy tissues.

**Results:**

The miniaturized combined DRS and AF spectroscopy system could accurately distinguish tumors from healthy liver with a sensitivity of 95% and a specificity of 96% from a sample size of N=10 patients and 52 measurements.

**Conclusions:**

We demonstrate the feasibility of miniaturizing a combined DRS and AF spectroscopy system for the classification of tumor and healthy tissues. This validation supports the feasibility and further development of a handheld, optically guided biopsy device.

## 1 Introduction

Colorectal cancer is the third most frequent type of cancer and the second most common cause of cancer-related death worldwide.^1^ Approximately 50% of patients with colorectal cancer present with liver metastases at initial diagnosis or develop liver metastasis during the course of the disease.^2^ Liver biopsy is an essential procedure for initial diagnosis and molecular analysis to determine the most appropriate treatment. However, even with imaging guidance methods, ∼15% of percutaneous liver biopsies are unsuccessful.^3^ It has been clearly demonstrated that increasing the number of needle passes per procedure improves diagnostic yield. However, increasing the number of needle passes also significantly increases the risk of complications.^4^ There is, therefore, an unmet need to increase the success rate of percutaneous liver biopsies without increasing the number of needle passes and possibly even decreasing the number of needle passes needed to obtain an adequate diagnostic yield.

One way to improve the diagnostic yield without increasing the number of needle passes is to ensure that the tumor tissue is recognized during the biopsy procedure. This can be achieved by integrating an optical tissue differentiation technique into the tip of a biopsy needle. When combined with the usual imaging techniques, these optical sensing modalities provide additional local information that can potentially improve the success rate of biopsies. Two optical methods that have been researched for liver lesion recognition are diffuse reflectance spectroscopy (DRS) and autofluorescence (AF) spectroscopy.^5^^–^^9^ DRS typically illuminates a sample with broadband visible and near-infrared (NIR) light and collects the diffusely scattered light with a fiber-coupled spectrometer at a specified distance from the source. DRS is capable of distinguishing among different types of tissues based on their distinct optical absorption and scattering properties. Typically, the level of absorption is determined by the composition of the tissue, whereas the extent of scattering is influenced by the tissue’s structural morphology.^10^ By contrast, AF spectroscopy measures the characteristic fluorescence spectra from endogenous fluorophores in tissues and can distinguish among different tissue types based on their fluorescence spectrum. These properties are determined by the molecular composition. Several endogenous fluorophores have been reported in liver tissues, including metabolic compounds such as FAD and NAD(P)H and other compounds related to liver function such as bile and bilirubin.^11^^,^^12^ Importantly, DRS and AF are both well suited for fiber coupling using a common excitation fiber and separate collection fibers. DRS requires a spatial separation between the emission and collection fibers, whereas the AF emission is much weaker and must be collected close to the emission fiber. Combining DRS and AF in one device allows the use of both diffuse optical and autofluorescent properties, thus increasing the discriminative potential.

For an optical biopsy device to help the operator improve the success rate of percutaneous biopsies, not only the device should be able to differentiate among different tissue types but also the design of the device should be easily incorporated into the current clinical workflow.^13^ For ultrasound-guided percutaneous liver biopsies, the operator should be able to hold the ultrasound with one hand while holding the biopsy device with the other. In addition, the room in which the procedure takes place should be large enough to accommodate the ultrasound machine.^4^ Therefore, it would be best to incorporate the optical design into the biopsy actuator and needle without the need for an additional trolley or device. The needle core for percutaneous liver biopsies should be no larger than 16 G (1.29 mm) in diameter, and the sampling notch should be designed to collect a sample with an intact core that is 0.8 to 1.0 mm in diameter and 20 mm in length.^4^

This study is researching the feasibility of an optical biopsy setup combining AF and DRS to discriminate tissue types. A fiber-optic needle probe with a small diameter, similar to an actual biopsy needle, is being tested on colorectal liver metastases in an *ex vivo* setting. Miniature spectrometers are being used to allow for potential future integration of the optical design into a smart biopsy device. Finally, the performance of a classification model discriminating tumor tissues from healthy liver tissues using only DRS, only AF, and a combination of DRS and AF was calculated. Importantly, this study demonstrates that DRS and AF data can be collected through optical fibers embedded in a fiber-optic needle probe and the optical signals resolved with mini spectrometers, thus providing an important validation of the technologies required for a handheld optically guided biopsy device.

## 2 Methods

In this prospective *ex vivo* study, DRS and AF measurements were taken on excised liver segments that had been sliced to visualize colorectal metastasis. Technical details are provided in the following of the optical setup, including the needle probe design, light sources, and spectrometers. To aid the understanding of the *ex vivo* liver measurements and associate the observed spectral features with physiologically relevant biomarkers, AF spectra were measured of the liver biomarkers bile and bilirubin in a liquid solution. From these measurements and values reported in the literature,^5^^–^^9^^,^^11^^,^^12^ features within the measured DRS and AF spectra were selected to discriminate healthy liver tissues from tumorous liver tissues. The measurements were subsequently classified using a machine learning algorithm, which utilized only DRS, only AF, and a combination of DRS and AF. Further details about the study procedure will also be provided.

### 2.1 Biomarker Characterization

The fluorescent emission properties of bilirubin and bile were investigated here under 415 nm excitation (Thorlabs, Newton, New Jersey, United States, M415L4) as a benchmark for measurements on tissues. The fluorescent emissions were collected in transmission using a fiber-coupled benchtop spectrometer (Wasatch Photonics, Morrisville, North Carolina, United States, WP-VISNIR-R2-IC, 400 to 1080 nm). Liquid samples were placed in transparent plastic cuvettes with a 10 mm path length, and the fluorescence was measured at an angle perpendicular to the excitation light. Bilirubin powder (bile pigment), Ox-bile, and dimethyl sulfoxide (DMSO) were purchased from Merck, Wicklow, Ireland. Ox-bile was dissolved in deionized water at a concentration of 10  mg/mL. Bilirubin powder was dissolved in DMSO to prepare a stock solution of 2  mg/mL, which was then diluted with deionized water to achieve a concentration of 0.2  mg/mL. The concentrations are selected to be in the physiologically relevant range.^14^

### 2.2 Optical Setup

**Fig. 1:**
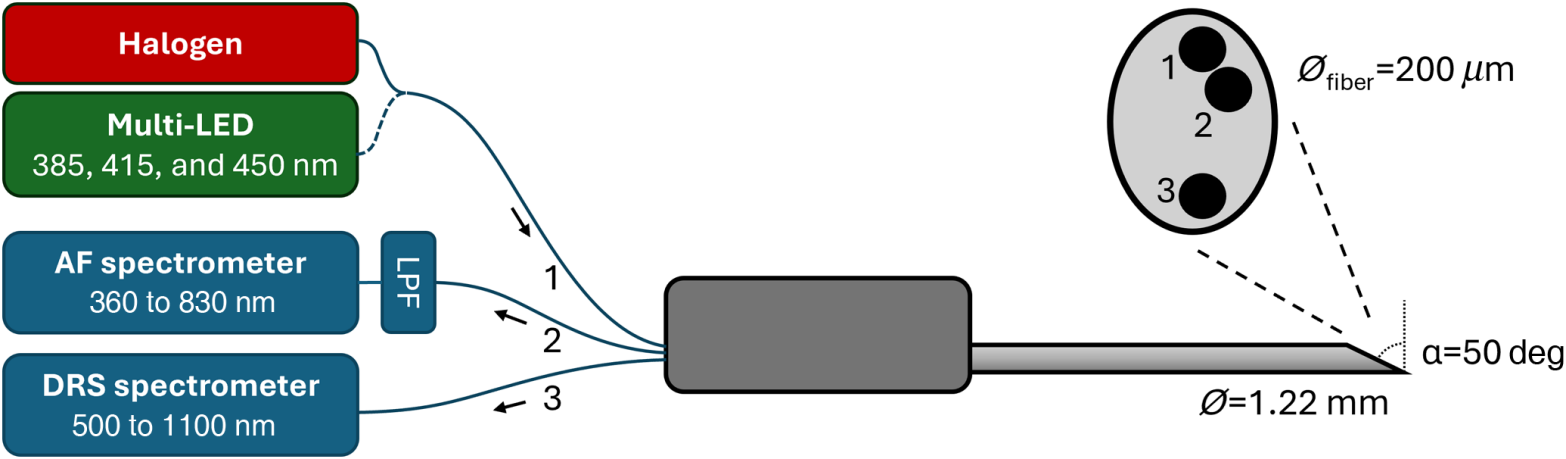
Schematic of the needle probe and fiber-coupled sources and sensors. The needle probe has three optical fibers positioned along the sloped vertical axis: the top-most fiber is used for illumination from either a broadband halogen source for DRS or the multi-LED source for AF. The AF collection fiber is positioned in contact with the emission fiber, whereas the DRS collection fiber is spatially separated from the emission fiber. The collection fibers are connected to two mini spectrometers with an in-line LPF to remove AF excitation crosstalk.

The optical system was designed so that future integration of all optical components into the biopsy handle is feasible. For the needle probe used in this study, the fiber separation was kept small to leave room for a biopsy sample notch. Similarly, mini spectrometers were used for both DRS and AF to allow for the future placement of them into the biopsy handle. A schematic overview of the setup can be seen in Fig. 1.

The needle probe had a diameter of 1.22 mm (∼18  G) and contained one emission fiber, and two detection fibers for DRS and AF, respectively. All fibers had a diameter of 200  μm and 0.22 NA, which was selected as the maximum fiber dimension that would fit inside the needle with the spatial separation required for DRS. The tip of the needle probe was beveled at 50 deg to optimize needle sharpness while minimizing total internal reflection when the fibers are in contact with tissues.^15^ The fibers run parallel with the needle and have the same bevel angle. The three optical fibers were positioned along the sloped axis to facilitate a sampling notch in future designs.^16^ The AF collection fiber was in contact with the emission fiber. By contrast, the DRS collection fiber had a spatial separation of ∼0.93  mm (core to core) to collect predominantly diffusely reflected light.

The system used two mini spectrometers covering the visible 360 to 830 nm and visible–NIR 500 to 1100 nm for AF and DRS, respectively (Ibsen Photonics, Farum, Denmark, PEBBLE VIS and PEBBLE VIS–NIR, $20\times 15\times 8\;{\mathrm{mm}}^{3}$). The integration time was 50 ms for DRS and 100 ms for AF, respectively. To effectively suppress crosstalk from the multi-light emitting diode (LED) AF excitation source while collecting the AF spectra, a long-pass filter (LPF) (Semrock, Rochester, New York, United States, BLP01-458R-25, 458 nm edge) in a fiber-coupled filter mount (Thorlabs, Newton, New Jersey, United States, FOFMS/M) was inserted before the spectrometer.

The system used separate illumination sources for DRS and AF. For DRS, the illumination source was a fiber-coupled halogen (Thorlabs, Newton, New Jersey, United States, SLS201L/M, 360 to 2600 nm) with a −132 mired color temperature balancing filter (Thorlabs, Newton, New Jersey, United States, FGT165M) to flatten the emission spectrum across the measurement range. For AF, a multi-LED source was used, consisting of three printed circuit board (PCB)-mounted LEDs (Thorlabs, Newton, New Jersey, United States, M385D2, M415D2, and M450D3) that are spectrally combined through dichroic mirrors (Edmund Optics, Barrington, New Jersey, United States, TECHSPEC Dichroic LPFs, 400 and 435 nm) and the output coupled to the multimode fiber. The average power for AF excitation was 2.1 mW (385 nm), 3.3 mW (415 nm), and 2.5 mW (450 nm), respectively. The detailed design of this light source is being communicated separately. The AF excitation wavelengths were selected to excite key biomarkers in the liver indicative of metabolism and liver function: 385 nm excitation is predominantly for detecting NAD(P)H, 415 nm excitation is for bile and bilirubin and porphyrin derivatives, and 450 nm is for FAD and bilirubin.^5^ The two excitation wavelengths related to metabolism are not reliable for the *ex vivo* tissue in this study, and the main focus is therefore on the nonmetabolic biomarkers and 415 nm excitation.

The system control and data collection were fully software-automated in LabView (National Instruments, Austin, Texas, United States). However, the source fiber had to be manually switched between the halogen and multi-LED sources for DRS and AF measurements in this study. This can easily be automated with an optical switch.

### 2.3 Patient Criteria

This *ex vivo* study complied with the Declaration of Helsinki and was approved by the Institutional Review Board of the Netherlands Cancer Institute/Antoni van Leeuwenhoek (Amsterdam, the Netherlands). All patients included in this study gave permission for noninvasive research based on their medical records and collected biological samples. Patients scheduled for liver resection of colorectal metastasis with a minimum diameter of 1 cm were included. Patients with liver metastasis from any other origin than colorectal and patients enrolled in other clinical trials were excluded.

### 2.4 Measurement Protocol

**Fig. 2:**
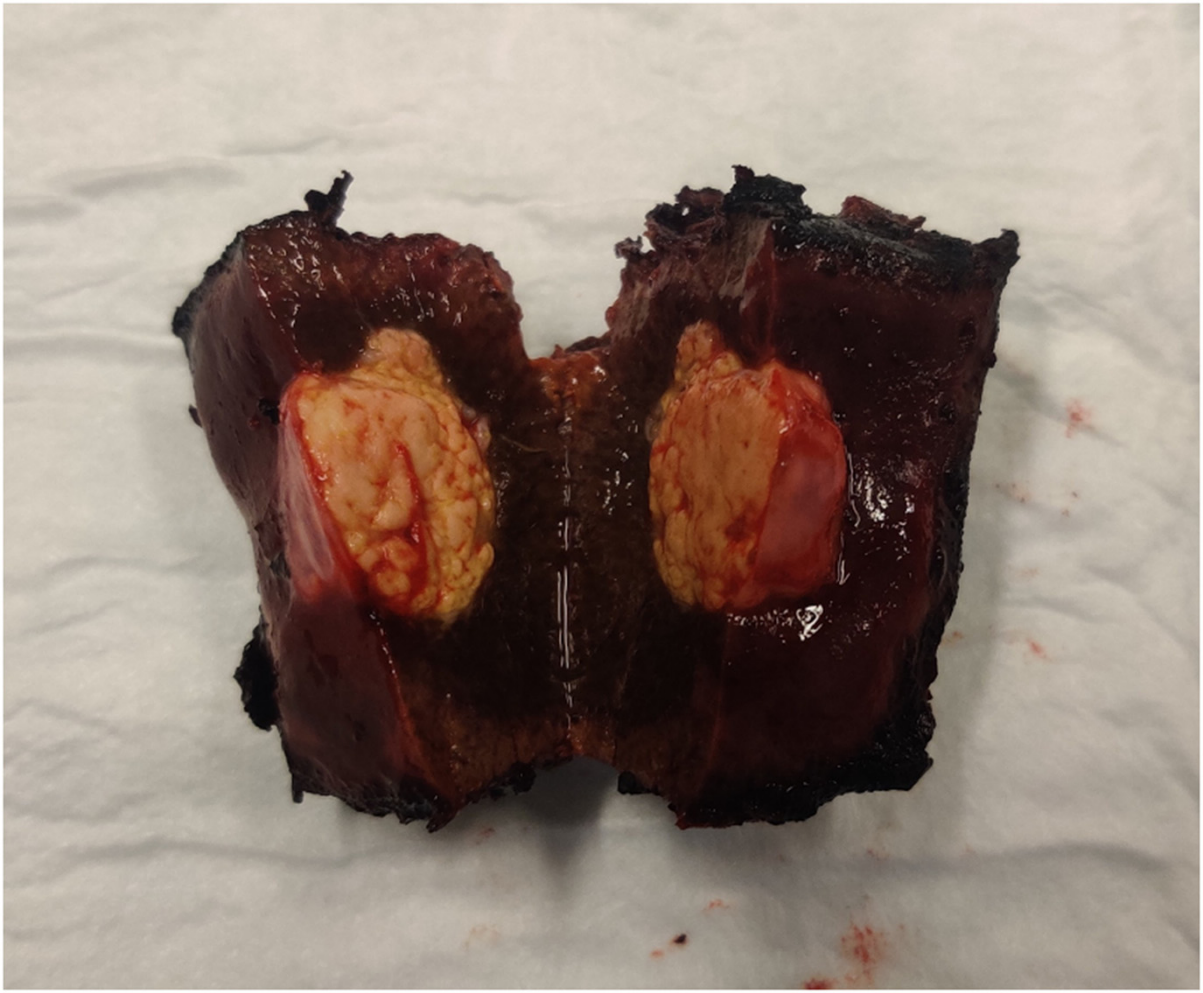
Photo of excised liver tumor that has been sliced through the middle. Tumor tissue has a yellowish appearance, whereas healthy tissue is of a dark red color.

Excised liver segments were brought to the histopathology department straight after surgery. Here, the pathologists sliced the excised liver segments so the tumor tissue was visible on the sliced surface. An example of such a sliced liver segment can be seen in Fig. 2, in which the tumor tissue has a yellowish appearance, whereas the healthy liver tissue is of a dark red color.

Measurements were conducted on these sliced liver segments with DRS and AF spectra recorded on both tumor tissue (yellow-colored tissue) and healthy tissue (red-colored tissue). Before commencing the measurements, the DRS system was calibrated using a “white” reference (Spectralon, Avantes, Apeldoorn, Netherlands, WS-2) and dark background measurement. The reflectance is calculated as

$$R=\frac{\text{Signal}-\text{Background}}{\text{Reference}-\text{Background}}.$$ (1)

A dark background was also captured for the AF system and subtracted from the fluorescence spectra. First, the DRS measurements were conducted with the needle probe’s emission fiber connected to the halogen light source. Depending on the tumor size, two to five DRS measurements of tumor tissues were taken in different locations, followed by two healthy measurements. All measurements were taken on liver segments that were sliced in half to expose the tumor tissues, thus allowing measurements on the tumor surface. The measured liver segments had a minimum thickness of 1 cm. Then, the AF measurements were conducted with the emission fiber connected to the multi-LED source. Measurements were taken at the same locations as the DRS measurements. All AF and DRS measurements were taken with the ambient light switched off. The optical measurements were performed on the surface of the tissue slices to ensure that the data were recorded on a well-defined region, i.e., tumor or healthy, but it has been validated that the recorded spectra are similar when piercing the tissue surface.

### 2.5 Data Analysis

A supervised machine learning model was used for the data analysis using MATLAB’s Classification Learner (MathWorks Inc., Natick, Massachusetts, United States). The aim was to distinguish tumors from healthy tissue based on acquired DRS and AF spectra. A linear discriminant analysis (LDA) with fivefold cross-validation was applied to the DRS and AF data separately and to the combined DRS and AF datasets.

To prevent overfitting, spectral bands were selected to calculate ratios for both DRS and AF, where the reflectance or spectral intensity was integrated over the selected range. For the classification of DRS data, two ratios were calculated from three spectral bands

$$\text{Ratio }1=\frac{\overset{¯}{R}[475-575\;\mathrm{nm}]}{\overset{¯}{R}[800-900\;\mathrm{nm}]},$$ (2)

$$\text{Ratio }2=\frac{\overset{¯}{R}[1100-1120\;\mathrm{nm}]}{\overset{¯}{R}[800-900\;\mathrm{nm}]},$$ (3)

where $\overset{¯}{R}$ is the measured reflectance averaged over the specified range. The range 475 to 575 nm is selected to capture the visible contrast between healthy and tumor tissues seen in Fig. 2, where the visible region is generally dominated by hemoglobin absorption. The range 1100 to 1120 nm is at the edge of lipid and collagen absorption peaks. The long wavelength side is limited by the detection range of the CMOS spectrometer. The range 800 to 900 nm is used for normalization as it has a stable absorption with no major absorption peaks.^6^^,^^17^

**Fig. 3:**
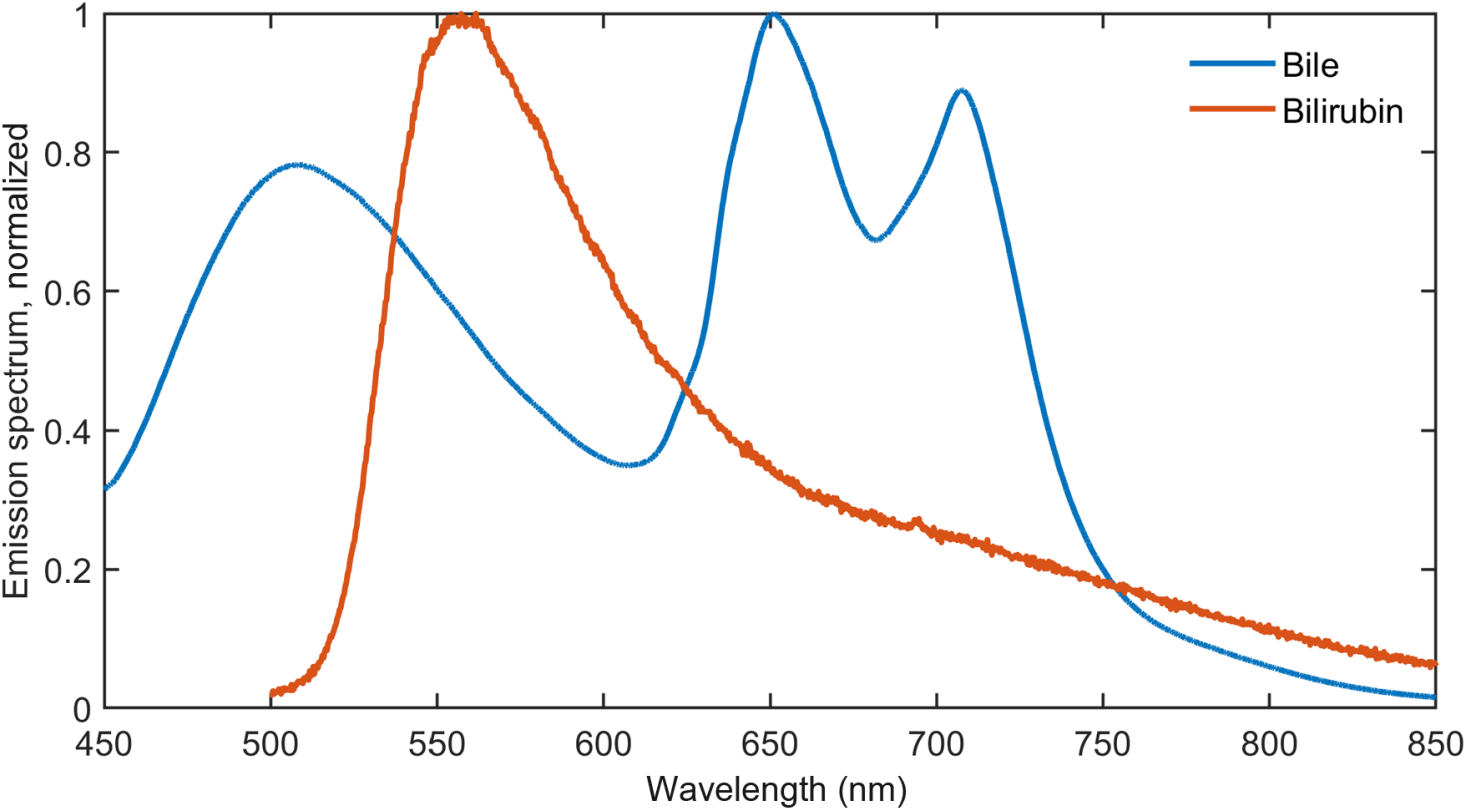
Emission spectra of bile and bilirubin using an excitation wavelength of 415 nm. Ox-bile was dissolved in deionized water to 10  mg/mL, and bilirubin was dissolved in DMSO and then diluted with deionized water to 0.2  mg/mL.

**Fig. 4:**
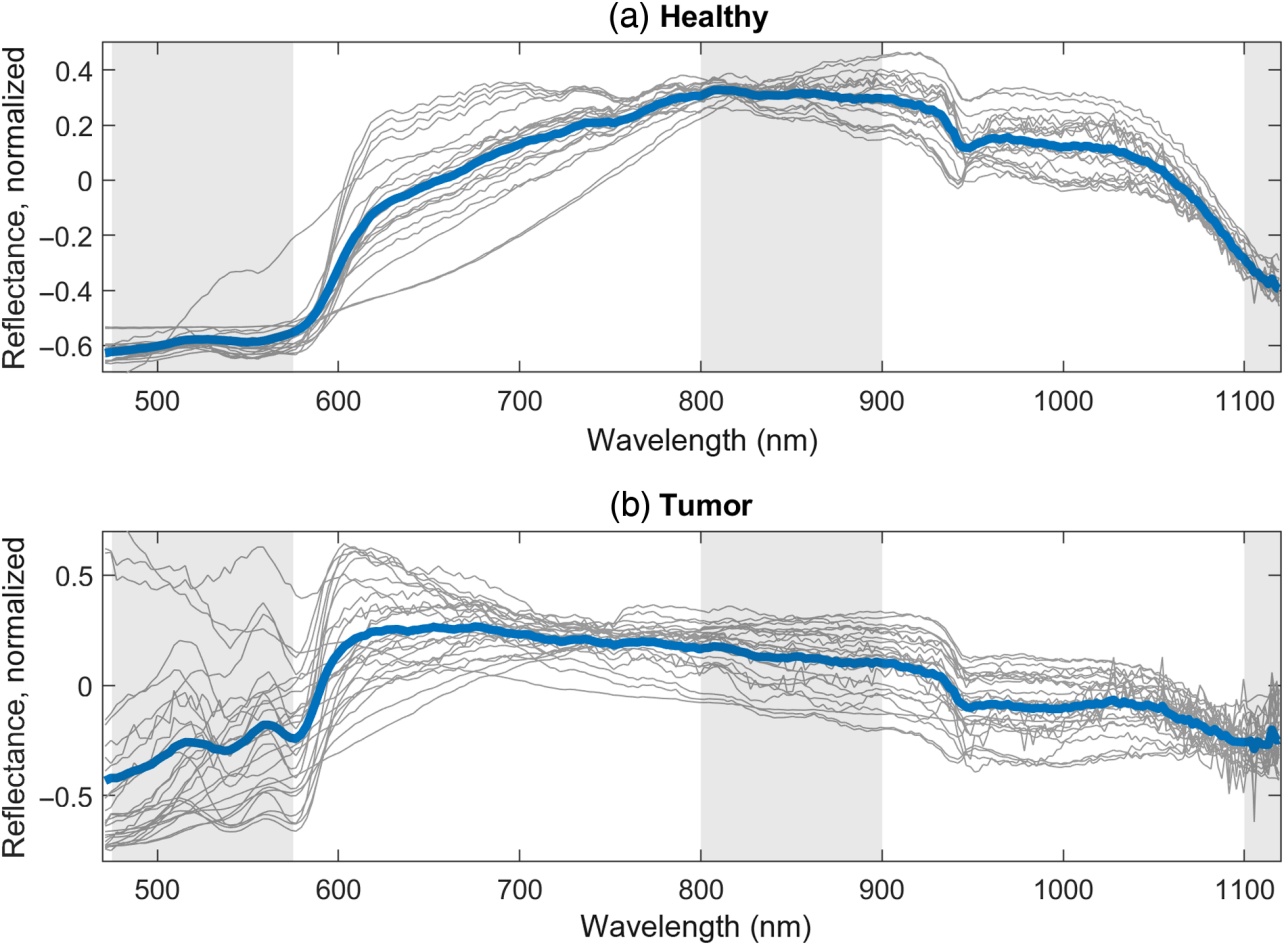
DRS measurements of the (a) 24 healthy and (b) 28 tumorous tissues. The thin gray lines are individual measurements, and the thick blue lines are the average of all measurements in each class. The spectra are mean-normalized for better visualization. The shaded gray areas mark the spectral regions used in the further data analysis.

**Fig. 5:**
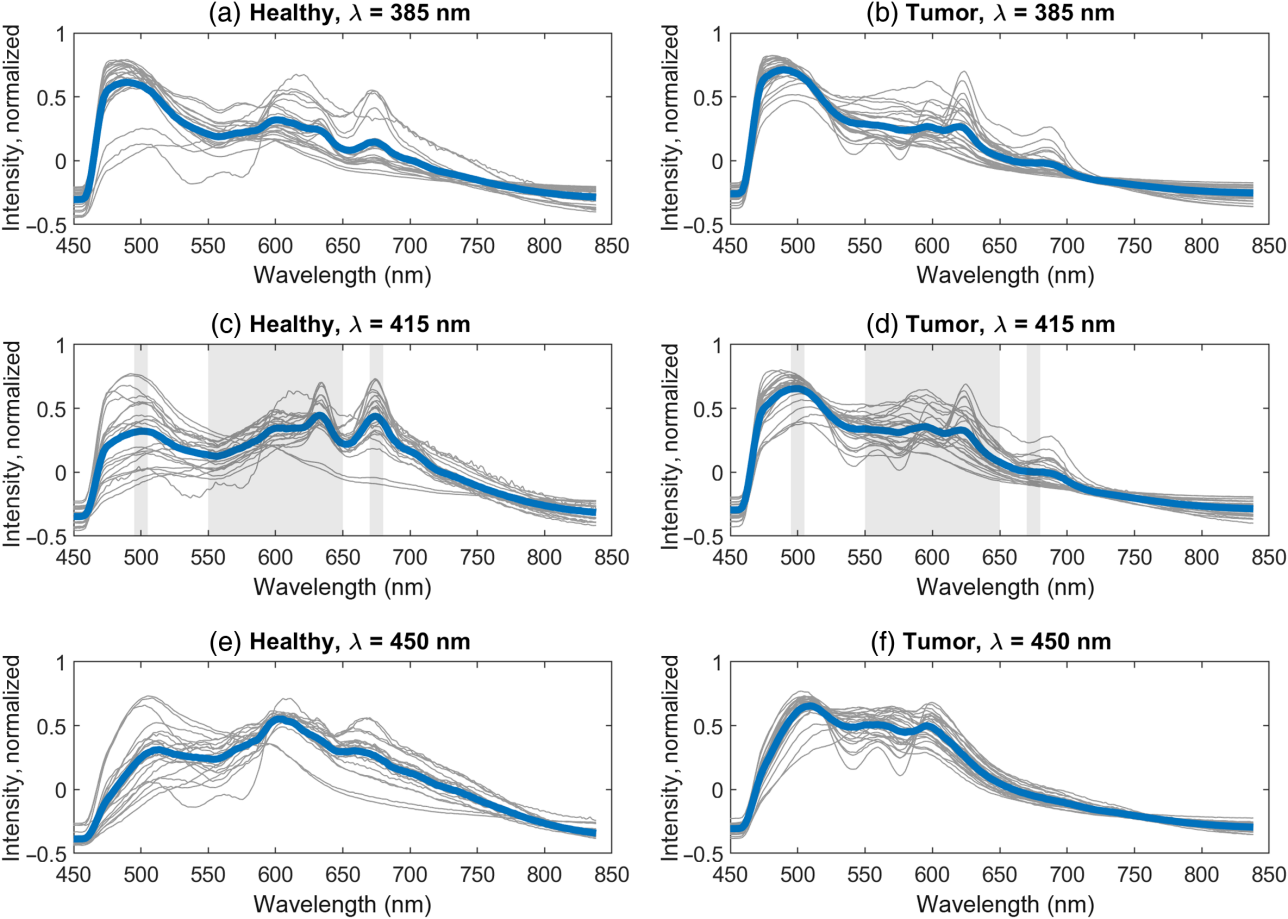
AF measurements of healthy and tumor tissues with excitation at (a) and (b) 385 nm, (c) and (d) 415 nm, and (e) and (f) 450 nm, respectively. Gray lines are individual measurements, and the thick blue lines are the average of all measurements. The spectra are background subtracted and mean-normalized for better visualization. The shaded gray areas in panels (c) and (d) mark the spectral regions used in the further data analysis.

The classification of AF data has been limited to the 415 nm excitation to detect bile and porphyrin as the other excitation wavelengths primarily target metabolic biomarkers. Here, the selected ratios were chosen based on the emission spectra of bile (see Fig. 3). The average intensity is calculated in two 10 nm wide bands centered at 500 and 675 nm; the former is the primary bile peak, and the latter is from porphyrin. The average value in a 100 nm wide band at 600 nm is used for normalization. The ratios were

$$\text{Ratio }3=\frac{\overset{¯}{I}[495-505\;\mathrm{nm}]}{\overset{¯}{I}[550-650\;\mathrm{nm}]},$$ (4)

$$\text{Ratio }4=\frac{\overset{¯}{I}[670-680\;\mathrm{nm}]}{\overset{¯}{I}[550-650\;\mathrm{nm}]},$$ (5)

where $\overset{¯}{I}$ is the background subtracted intensity counts averaged over the specified range. The selected spectral bands are marked in the AF and DRS measurements (Figs. 4 and 5).

## 3 Results

### 3.1 Biomarker Characterization

Unlike FAD and NAD(P)H, the research on autofluorescence of bile and bilirubin is relatively limited. The measured fluorescence spectra of bile and bilirubin in solution are shown in Fig. 3. It is observed that the bile emission spectrum has three distinctive peaks at wavelengths at ∼510, 650, and 710 nm, whereas bilirubin has a peak at 560 nm. The characteristic double peak observed in the bile emission spectrum can be ascribed to porphyrin derivatives in the ox-bile and expected in liver tissues.^12^ Previous reports in the literature have shown that bilirubin in PBS solution emits in the 500 to 600 nm region with peaks at 520 and 570 nm from 366, 420, and 465 nm excitation,^11^ and bile from rat liver emits in the 400 to 490 nm region from 366 nm excitation.^12^ In contrast to previous reports, the 560 nm bilirubin peak is not immediately visible in the bile emission spectrum in Fig. 3, although it may explain the small shoulder. It should also be noted that at these concentrations, the bilirubin emission is more than 10 times weaker than the bile emission. There are a few factors that can account for some of the differences between this work and previous reports, in particular the different excitation wavelengths, different solvents, and bile from a different animal. However, the spectral peaks from the bile emission spectrum are observed in the *ex vivo* and therefore used in the subsequent analysis.

### 3.2 Dataset

A total of N=10 patients met the inclusion criteria, resulting in 52 DRS measurements and 52 AF measurements. Out of these, 24 measurements were of healthy liver tissues, and 28 were of tumorous tissues.

The average DRS spectra for tumor measurements and healthy measurements can be seen in Fig. 4. Because of the variation in reflectance among measurements, the spectra are mean-normalized for better visualization. This variation is mainly due to the long and thin needle probe, which meant that it was difficult to keep the contact pressure the same for all measurements. Notwithstanding, there are clear differences between the healthy and tumor measurements: the reflectance in the visible range is generally much higher for tumor tissues, as expected from the photograph in Fig. 2. The characteristic hemoglobin spectral features are also observed in the 550 to 600 nm range. The variation in this range is much higher for tumor tissues, which is likely because of blood on the pale tumor tissue from when it was sliced. For the NIR range above 1050 nm, the reflectance is notably lower for healthy tissues due to lipids and collagen. Overall, there is a larger variation among individual measurements for tumor tissues, which suggests a larger variation in tissue composition than for healthy tissues. In both datasets, there appear to be a few outliers that may have been the result of different neoadjuvant treatment strategies among patients.

The AF spectra for healthy and tumor locations can be seen in Fig. 5 for all three excitation wavelengths. Similar to the DRS results, the figures show both individual measurements and the average for each tissue class. Generally speaking, the 385 nm source will predominantly excite NAD(P)H with a broad emission at 450 nm, and the 450 nm source will excite FAD with a broad emission at 540 nm.^5^ In contrast to these metabolic biomarkers, the 415 nm source excites bile and bilirubin, as seen in Fig. 3, making it the most relevant for the *ex vivo* samples studied here. The 385 and 450 nm sources are mainly expected to bring value when going to *in vivo* measurements and are also included in this prospective study.

For the 385 nm excitation in Figs. 5(a) and 5(b), there are no clear spectral features to differentiate between healthy and tumor tissues. It should be noted that the 458 nm LPF inserted before the spectrometer filters out the NAD(P)H emission peak but not the long tail. The 415 nm excitation in Figs. 5(c) and 5(d) shows several distinctive spectral features: the broad 500 nm peak from bile appears more prominent in tumor tissues, whereas the double porphyrin peaks are observed for healthy tissues. There are no obvious peaks at 560 nm from bilirubin. The emission above 600 nm is generally stronger in healthy tissues. These features are used for classification. Finally, the 450 nm excitation in Figs. 5(e) and 5(f) also shows spectral differences between healthy and tumor tissues, in particular, a stronger emission above 600 nm in healthy tissues similar to the 415 nm excitation.

It should be noted that the AF fluorescence is influenced by the scattering and absorption, which is very different for healthy and tumor tissues, as seen in Figs. 2–4. For example, the hemoglobin peaks seen in the DRS spectra ∼540 to 580 nm for tumor tissues are also seen in the AF spectra.

### 3.3 Classification

The DRS and AF measurements presented above show spectral features that can differentiate healthy and tumor tissues. For DRS, the ratios are selected to capture the visible contrast between healthy and tumor, and the lipid and collagen absorption, respectively. The AF ratios are based on the primary emission band for bile and the porphyrin peak in the red region, respectively. As described previously, a ratiometric method was used to normalize spectral variations, and the full DRS and AF spectra were reduced to two ratios to avoid spectral overfitting. An LDA model was applied to differentiate between the healthy and tumor tissues using the calculated spectral ratios. Table 1 summarizes the classification results for DRS alone, AF alone, and DRS and AF combined. The classification results for DRS and AF alone are influenced by a few outliers, whereas the combined DRS and AF classification is seen to improve the sensitivity and specificity as expected.

**Table 1:** Classification model results for DRS and AF results and for the combined DRS and AF datasets using a linear discriminant analysis with fivefold cross-validation. The analysis is based on the four spectral ratios defined in the text.

	Sensitivity (%)	Specificity (%)
DRS	95	81
AF	82	96
DRS and AF	95	96

**Fig. 6:**
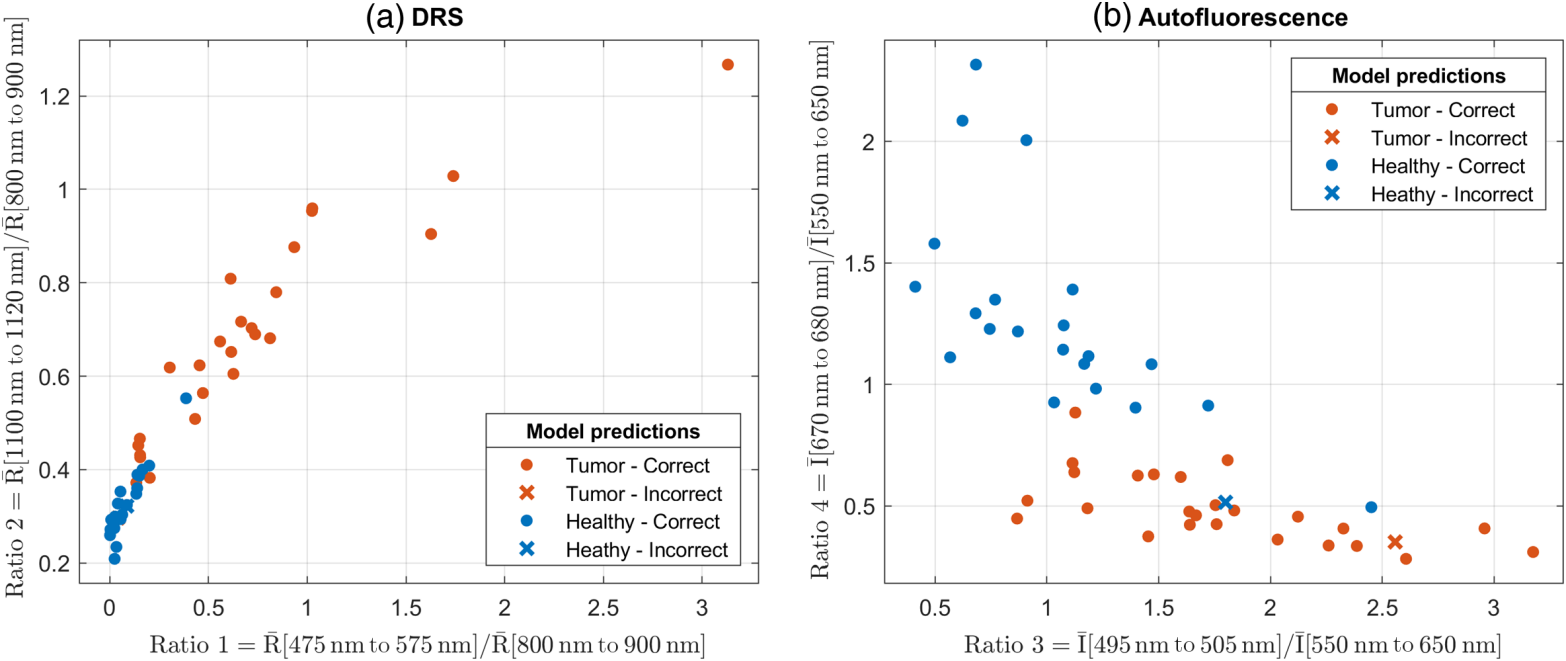
Classification scatter plots for (a) DRS reflectance ratios and (b) AF intensity ratios. The model predictions are for the combined DRS and AF models.

The classification scatter plot is shown in Fig. 6 with the predictions from the combined DRS and AF model. Overall, the selected spectral ratios are seen to separate the two classes well for both DRS and AF although a few incorrectly classified data points remain.

## 4 Discussion

In this study, the feasibility of an optical biopsy setup combining AF and DRS to discriminate tissue types was assessed. Miniature spectrometers and a fiber-optic needle probe with a diameter similar to an actual biopsy needle (Ø 1.22 mm) were used to allow for future integration of the optical design into a biopsy actuator with the ultimate goal of improving the diagnostic yield and overall success rate of percutaneous biopsies. It is important to note that this technique is part of a broader diagnostic workflow. Initially, liver lesions are detected via magnetic resonance imaging (MRI) or computed tomography (CT), followed by confirmation with an ultrasound-guided biopsy. The optically guided biopsy procedure will complement, not replace, ultrasound—its role is to improve the accuracy and success rate of targeting lesions.

Optical tissue recognition using DRS and/or AF measured through optical needle probes has received significant attention. For example, the Probea system measures AF through a 25 G needle and has been used for tumor detection in breast^18^ and lung.^19^ Braun et al.^20^ combined DRS and AF in a 19 G needle with 7 optical fibers for *in vivo* lung carcinoma detection. In addition, a subset of the authors of this work, led by Prof. Ruers, has investigated DRS and AF for breast and liver cancer with needle-based systems.^6^^,^^8^^,^^16^^,^^17^^,^^21^ Dremin et al.^7^ demonstrated DRS and AF sensing with a 17.5 G percutaneous needle probe with 10 optical fibers to differentiate healthy and tumor liver tissues in an *in vivo* study. It has thus been demonstrated that optical sensing can accurately classify tissue types through needle probes across a wide range of applications. However, previous studies have used large and often high-sensitivity spectrometers to record and analyze the optical signals that can not be integrated into a handheld device. The system presented here is based on mini spectrometers with a footprint that can be integrated into the handle of a typical biopsy device. Moreover, the needle probe has been designed to accommodate a side-notch to collect a tissue sample. This is possible because the combined DRS and AF spectroscopy is performed using only three optical fibers. Integrating the optical sensing into the biopsy needle ensures that the sample collection and optical measurements are from the same tissue volume. Importantly, it has been demonstrated that optical fibers can be routed under a biopsy sample collection notch.^16^ By contrast, using a separate probe for the optical measurement complicates the clinical workflow and adds uncertainty if the biopsy sample is collected from the same tissue volume as the optical measurement.

The long, thin needle probe used here meant that it was challenging to maintain the same contact pressure for all measurements, even though all measurements were performed by the same person. This inevitably increased the variability among measurements as seen in the recorded DRS and AF spectra. Spectral normalization made some improvements in visualizing and comparing the data, and the spectral classifiers are all ratiometric for this reason. However, it is very encouraging that the optical tissue classification works well despite these variations, which demonstrates the robustness of using optical sensing in a biopsy setup. It is expected that the data collection will improve for biopsy measurements with the needle piercing the tissue *in vivo* instead of being held against the tissue surface. This will eliminate any surface effects from, e.g., blood and ensure a more consistent contact pressure. The time from the tissue resection to the optical measurement will vary for *ex vivo* measurements and lead to variations in the tissue state, which will also be eliminated with *in vivo* measurements. The measurements in this study were all recorded with the same needle probe. There were no signs of degradation, which demonstrates the suitability of using optical fibers for soft tissue measurements. It should be noted that a biopsy needle will always be disposable.

It should also be noted that there is a trade-off between sensitivity and specificity and that the results presented here are based on a relatively small dataset (N=10 patients). The classification results are optimized to give the best classification accuracy although it may be preferable to adjust the model for sensitivity to minimize the number of false-negative predictions. False-positive results will generally be reassessed in histopathology. Overall, the results are very encouraging and may be sufficient for some applications although a larger number of samples are required to fully validate the technology and improve the classification algorithms.

The fiber placement within the needle tip is a key design constraint: the AF collection fiber should be very close to the excitation fiber, whereas the DRS collection fiber should have a spatial separation to collect diffusely scattered light. The fibers used here have a core diameter of 200  μm and 0.22 NA to give a good optical throughput and a spatial separation of close to 1 mm for the DRS fibers. Although a larger distance is desirable to collect only diffuse photons, the results presented here show that a submillimeter distance is sufficient to provide good optical contrast between healthy and tumor tissues. Alternative fiber configurations could be considered in the future, especially for applications requiring a smaller needle diameter. For example, DRS and AF can potentially be measured with just two fibers if the AF signal is collected through the emission fiber. The excitation light and AF signal can then be separated through, e.g., an LPF or using a double-clad fiber.^22^ This would also benefit from a better spatial overlap between the excitation and AF collection fiber. It is, however, encouraging that the results presented here show that AF spectra can be collected through a fiber placed next to the emission fiber.

The main aim of this work was to demonstrate the feasibility of measuring DRS and AF through a needle probe with a view toward the integration into a handheld device. It is, therefore, very promising that the quality of the measured DRS and AF spectra enables accurate classification of tissues. However, one downside of CMOS-based spectrometers is the long integration time required, especially for AF, which typically needs 100 ms for each excitation source. This can, for example, be improved with highly sensitive SiPM-based AF detectors, as demonstrated by Grygoryev et al.,^23^ which further improves the correction for ambient light. For DRS, it might be advantageous to measure further in the NIR region above 1000 nm to capture the fat and water contents,^6^^,^^17^ but this will require more expensive InGaAs-based spectrometers.

To fully integrate the DRS and AF systems into the handle of a biopsy device, further miniaturization is required, especially of the light sources. The results of this study suggest that all three AF excitation sources are unlikely to be needed. However, the AF spectra are expected to be different for *in vivo* measurements, where the metabolic biomarkers are more relevant. This will be investigated further in future studies. It may also be possible to measure both DRS and AF with a single spectrometer, which would reduce the number of components in the biopsy handle. It is our view that the clinical need for optically guided biopsy is best met with a handheld and untethered device, which requires full integration of sources and detectors, as well as wireless communications. This will be explored in future work.

The AF excitation wavelengths were selected to excite key biomarkers in the liver indicative of liver function and metabolism, in which 385 nm excitation was predominantly chosen for NAD(P)H; 415 nm for bile, bilirubin, and porphyrin derivatives; and 450 nm for FAD and bilirubin.^5^ This study was conducted in an *ex vivo* setting, where the metabolic biomarkers NAD(P)H and FAD may be present in the liver tissues but cannot be expected to change. The analysis was therefore limited to bile and porphyrin using only data from 415 nm excitation in the LDA classification model. Bile and porphyrin were chosen as biomarkers because these agents are present in healthy liver tissues and are expected to be less present in liver metastases from colorectal origin. However, comparing the results in Figs. 5(c) and 5(d) to the emission spectra of bile in Fig. 3 partially contradicts this theory. Here, the broad 500 nm peak from bile appears to be more prominent in tumor tissues compared with healthy tissues. The porphyrin double peaks are more clearly observed in the spectra from healthy tissues as expected. Therefore, it is unlikely that the more prominent 500 nm peak in tumor tissues can be fully attributed to bile, and there can easily be overlap with other biomarkers in that wavelength range.^5^ It should, however, be noted that the absorption in this spectral region is dominated by oxy- and deoxyhemoglobin. Finally, it should be emphasized that the AF spectra are mean-normalized to correct for measurement variations such as contract pressure. It was found that the absolute fluorescence intensity was comparable for healthy and tumor tissues. Despite this contradiction to the theory that bile would be less detectable in tumor tissues ∼500  nm, the LDA classification model was still able to correctly classify tumor measurements and healthy measurements with a sensitivity of 82% and a specificity of 96% (AF only). These numbers are likely to increase when adding metabolic biomarkers from NAD(P)H and FAD in an *in vivo* setting. It is also noteworthy that there is no clear observation of a bilirubin peak ∼560  nm as expected from the biomarker characterization in Fig. 3. This can be explained by the very weak emission from bilirubin relative to bile observed in the characterization. It should be emphasized that the micro-environment directly affects the AF absorption and emission spectra, which means that the emission in solution generally will be different from the emission in tissues. This has previously been shown for bilirubin in rat liver.^11^ This can explain the observed wavelength shift of the porphyrin emission peaks in tissues and possibly also the 500 nm peak in tumor tissues. The results presented here suggest that a complete analysis using, e.g., spectral fitting, requires further investigation and must include more biomarkers than just bile and porphyrin.

## 5 Conclusion

In this study, the feasibility of a handheld biopsy tool using a combined DRS and AF spectroscopy system for differentiating between healthy and tumorous liver tissues *ex vivo* was demonstrated. By utilizing a needle probe similar in diameter to standard biopsy needles and miniaturized spectrometers, promising classification results were achieved with a sensitivity and specificity of 95% and 96%, respectively, when combining DRS and AF data. This validates the technologies necessary for a handheld optically guided biopsy device. Future research should concentrate on validating the technique in an *in vivo* setting and implementing it in a clinical setting.

## Data Availability

Data underlying the results presented in this paper are not publicly available at this time but may be obtained from the authors upon reasonable request.
